# Synthesis of a potassium capped terminal cobalt–oxido complex†

**DOI:** 10.1039/d4cc03014a

**Published:** 2024-08-08

**Authors:** Sophie W. Anferov, Alexandra Krupinski, John S. Anderson

**Affiliations:** a Department of Chemistry, University of Chicago 929 E. 57th Street Chicago IL 60637 USA jsanderson@uchicago.edu

## Abstract

An unusual example of a potassium capped terminal cobalt–oxido complex has been isolated and crystallographically characterized. The synthesis of [^*t*Bu,Tol^DHP]CoOK proceeds from a previously reported parent compound, [^*t*Bu,Tol^DHP]CoOH, *via* deprotonation with KO^*t*^Bu. Structural and electronic characterization suggest a weakly coupled dimer in a distinct seesaw geometry with a Co(iii) oxidation state and a non-innocent radical ligand.

## Abstract 2

An unusual example of a potassium capped terminal cobalt–oxido complex has been isolated and crystallographically characterized.

## Graphical abstract

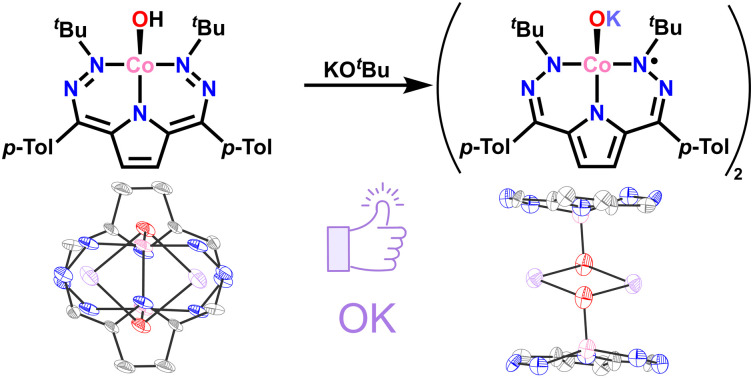

Transition metal–oxo compounds are widely studied for the role they play in both natural and synthetic systems.^1^ They are central intermediates in a wide range of oxidative transformations including oxygen transfer and C–H activation.^1–3^ In biological systems, key oxo complexes are present in the active sites of enzymatic structures such as cytochrome P450 and photosystem II. These systems have inspired study on various synthetic complexes which can similarly facilitate or model oxidative reactivity.^4^ Such complexes are commonly synthesized with mid-transition metals (*i.e.* Fe and Mn) both for their biological relevance but also for their synthetic accessibility and precedent.^1^ However, oxo complexes of later transition metals (groups 9–11) are more challenging to access due to the increasing number of antibonding electrons which disrupt M–O bonding. Therefore, stabilization of late transition metal–oxo complexes frequently requires symmetry changes away from octahedral geometries to stabilize metal–oxygen bonding and avoid running up against the “oxo wall.”^1,5,6^

High d-electron counts in the absence of stabilizing geometry changes result in weakened M–O bonding and complexes that are best thought of as oxidos due to O-localized lone pairs and charge. This is a generally unfavorable scenario, and terminal oxido complexes are expected to be highly reactive. In the absence of significantly stabilizing π-bonding, other methods must be employed to isolate these complexes. Indeed, there are several elegant examples in the literature where formally singly bonded terminal oxidos can be stabilized through secondary coordination sphere hydrogen bonding (H-bonding).^7–12^ Notably, Borovik and coworkers isolated a singly bonded Fe(iii)–oxido complex, [Fe(iii)H_3_buea(O)]^2−^, stabilized *via* a hydrogen bonding cavity around the oxygen atom.^8,9^ Subsequently, Fout and coworkers isolated another singly bonded Fe(iii)–oxido complex, [N(afa^Cy^)_3_Fe(iii)(O)](OTf), stabilized *via* a separate H-bonding framework (Scheme 1).^10–12^

**Scheme 1:**
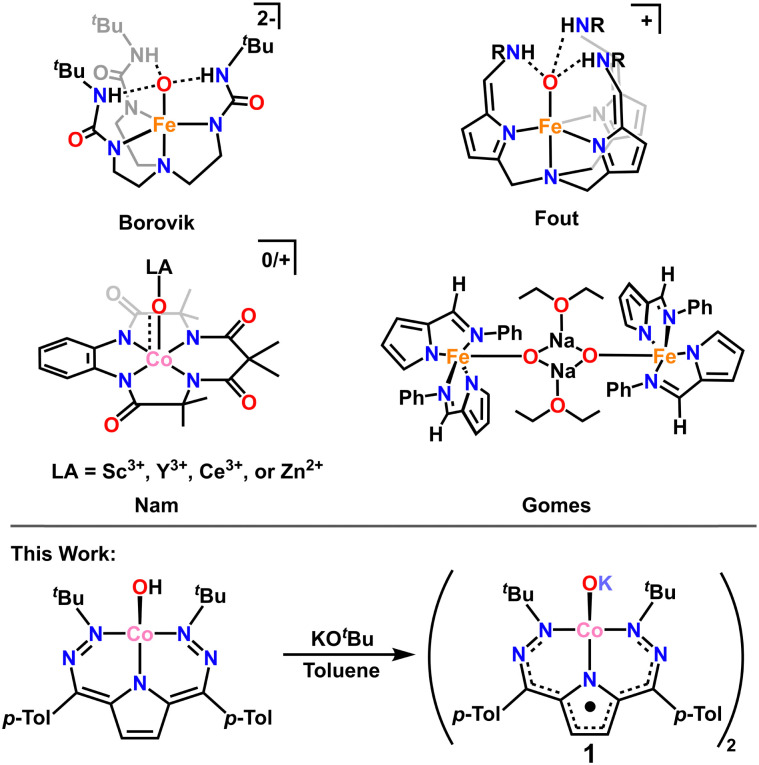
Top: Examples of previously reported metal oxidos stabilized by hydrogen bonding or Lewis acid interactions. Bottom: Synthesis of 1 from [^*t*Bu,Tol^DHP]CoOH.

An alternative method of stabilizing metal–oxygen bonds, and one that is employed in photosynthesis,^13^ is the use of Lewis acid stabilizers. There are several examples where Lewis-acidic (Sc^3+^ and Ce^3+^) metals have been used to stabilize Co–oxo complexes (among other M–oxo complexes, Scheme 1).^14–22^ Still, most of these examples are in high (>3) oxidation states and have some degree of metal–oxygen multiple bonding, unlike the previously mentioned H-bonding examples. Stabilization of metal–oxygen bonds by alkali metals and alkaline earth metals is an even rarer sub-category of Lewis acid stabilization. Jones and coworkers have reported the use of lithium^23^ and Borovik and coworkers have reported the use of calcium^24^ in stabilizing Co–hydroxide complexes. However, there is only one crystallographically characterized example of an alkali metal capped oxido complex from Gomes and coworkers who isolated and characterized a sodium capped Fe(ii)–oxido complex.^25^

In this work we report the first example of a Lewis acid stabilized Co–oxido, with potassium as the stabilizing Lewis acid. The complex [^*t*Bu,Tol^DHP]CoOK (1) was synthesized from the previously reported hydroxide—[^*t*Bu,Tol^DHP]CoOH—*via* direct deprotonation (^*t*Bu,Tol^DHP: 2,5-bis((2-(*tert*-butyl)hydrazineylidene)(*p*-tolyl)methyl)-1*H*-pyrrole).^26,27^ Complex 1 has been characterized by single-crystal X-ray diffraction (SXRD), high resolution mass spectrometry, and electron paramagnetic resonance (EPR), UV-vis, infrared (IR), and nuclear magnetic resonance (^1^H NMR) spectroscopies. The combination of these techniques reveals that 1 has an unusually distorted geometry and a formally Co(iii) electronic structure with a DHP ligand radical. Isolation of this compound also enables the experimental bracketing of the p*K*_a_ of the Co-bound hydroxide motif which can be extrapolated to <17, and likely ∼12.5 in water. These findings shed further light on the bonding and structure of late transition metal oxo/oxido complexes and provide a rare opportunity to obtain experimental acidity data for these species.

The oxido complex [^*t*Bu,Tol^DHP]CoOK (1) can be synthesized through the addition of 1–5 equivalents of KO^*t*^Bu (due to limited solubility) as a slurry to a dark purple solution of [^*t*Bu,Tol^DHP]CoOH in toluene (Scheme 1). The solution is stirred for 1 hour until a homogeneous dark purple solution is obtained. Drying and extraction provides 1 as a purple solid. The absence of an O–H stretch can be verified by the IR spectrum of this complex which confirms the deprotonation of the starting –OH moiety (Fig. S15, ESI†). Crystals of 1 can be grown out of a concentrated petroleum ether solution at −35 °C. SXRD analysis on dark purple needles shows a dimeric structure with a four-coordinate Co center and an O ligand in a roughly seesaw geometry (Fig. 1). The O ligands in the dimer are bridged with two K^+^ cations in a four-membered ring. Using the compound's N1–M–N5 and N3–M–O bond angles, the *τ*_4_ and the *τ*_4_′ values can be determined as 0.731 and 0.602 respectively. These values put complex 1 closest to a seesaw geometry 
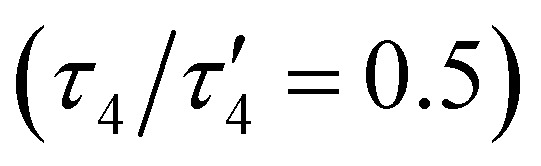
.^28,29^

**Fig. 1:**
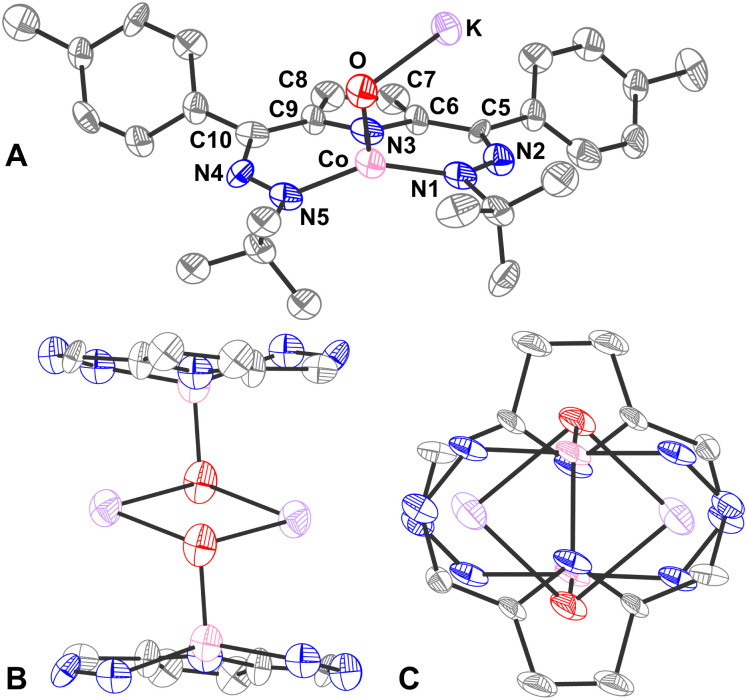
(A) SXRD structure of 1. (A) One half of the dimeric unit with the full DHP ligand. (B) A side view focusing on the O_2_K_2_ core with peripheral groups omitted. (C) A top view showing the stacked interactions with the K cations with peripheral groups omitted. Co (pink), N (blue), C (grey), O (red), K (violet). Ellipsoids at 50% and hydrogens omitted for clarity.

Comparison of the bond lengths of 1 with the parent hydroxide complex and previously reported metal–oxidos reveals some unusual geometric trends (Table 1). In contrast to metal–oxo complexes with multiple bonding that results in shorter M–O interactions, there is a significant elongation of the Co–O bond of 1 to 1.96(1) Å from the 1.825(2) Å length observed in the parent Co–OH. This M–O bond distance is also long when compared to Borovik's and Fout's Fe(iii)–O complexes where the Fe–O distances are 1.813(3) Å and 1.8079(9) Å respectively. The bond is also longer than Nam's Lewis acid stabilized compounds, [(TAML)Co(iv)(O)(M^*n*+^)], though this is unsurprising as those compounds are proposed to have partial double bond character.^18^ Perhaps more surprisingly, the Co–O bond length in 1 also lies outside the general range of other Co–O single bonds from the literature (1.784(3) Å–1.921(3) Å)^7,18,19,30,31^ as well as reported Co–O bond lengths of diamond core (Co(iii)–O)_2_ compounds (1.769(1)–1.832(5) Å).^32^ This observed bond elongation is likely attributed to stronger π-repulsion between the O lone pairs and the Co d-electrons. This π-repulsion is putatively higher due to the weaker acidity of the alkali metal bonded to the oxygen *versus* the proton in the corresponding hydroxide complex [^*t*Bu,Tol^DHP]CoOH. This hypothesis is supported by the similar bond length of Gomes’ previously reported Fe(ii)–O complex, 1.973(5) Å, which is also presumably elongated by the sodium ion's weak Lewis acidity. An interesting conclusion from the longer length of the Co–O bond in 1 is the comparatively weaker stabilization provided by alkali metal Lewis acids in contrast with the hydrogen bonding scaffolds employed by Borovik, Fout, and others.

Selected bond lengths (Å) and angles (°) of (1) and related complexes

**Table:** 

	1	[DHP^2−^] Co^III^OH^26^	Fe^III^–O Borovik^8^	Fe^III^–O Fout^11^	Fe^II^–O–Na Gomes^25^
M–O	1.96(1)	1.825(2)	1.813(3)	1.8079(9)	1.973(5)
O–M′1(Li/Na/K)	2.58(1)	—	—	—	2.263(6)
O–M′2(Li/Na/K)	2.64(1)				2.289(6)

**Table:** 

	1	[DHP^2−^] Co^III^OH^26^	[DHP^2−^] Co^II^(MeCN)^33^	[DHP^1−^] Co^II^OTf^33^
N1–N2/N4–N5	1.38(2)	1.306(3)	1.325(5)	1.273(10)
	1.32(2)	1.302(3)	1.320(5)	1.249(10)
C5–C6/C9–C10	1.44(2)	1.390(4)	1.402(6)	1.387(13)
	1.42(2)	1.383(4)	1.399(6)	1.392(13)
C7–C8	1.39(2)	1.349(4)	1.351(6)	1.315(13)
N1–M–N5	149.1(5)	162.4(1)	160.51(12)	178.1(3)
N3–M–X (O/N)	107.4(5)	143.5(1)	114.32(13)	113.152
				105.240

The effect of the alkali metal can further be contextualized by comparison with Jones’ hydroxide complex which has a similar “diamond-like” core. All three complexes have an O–M′ (M′ = Li/Na/K) bond about 2 Å long.^23^ Among these bonds, the Li–O bonds are shortest, followed by the Na–O bonds and K–O bonds which is consistent with the increase in ionic radii of each alkali metal. Further comparisons among the complexes’ geometries cannot be made because of different coordination environments around the transition metal centers, but DFT calculations predict similar periodic trends in the O–M′ distance for 1 (Fig. S24, ESI†).

The structural parameters of 1 also provide insight into its electronic structure. It should be noted that while [^*t*Bu,Tol^DHP]CoOH is formally a Co(ii) complex, it is more accurately considered with contributing Co(ii)/Co(iii) resonance structures with partial ligand radical character. In fact, [^*t*Bu,Tol^DHP]CoOH is closest to a Co(iii) oxidation state, as indicated by diagnostic changes in bond lengths when compared to clear-cut examples of a Co(ii)DHP^1−^ complex ([^*t*Bu,Tol^DHP]CoOTf) and Co(ii)DHP^2−^ complex ([^*t*Bu,Tol^DHP]Co(MeCN)) as standards.^33,34^ The metal–ligand redox-tautomerism observed in the structures of these complexes has been previously discussed.^33^

Comparison of the DHP ligand bond lengths in 1 with [^*t*Bu,Tol^DHP]CoOH reveals additional significant distortions. While the parent hydroxide compound geometry lies closer to square planar – with a *τ*_4_ of 0.38 and 
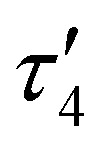
 of 0.33 – exchanging the H^+^ for K^+^ skews the complex towards a tetrahedrally distorted seesaw. This effect plausibly arises from potassium ion interactions with the π system on the DHP metallacycle. Such bond elongation from K^+^ has been previously observed, for instance, in work by Holland and coworkers.^35^ Further evidence of perturbative alkali cation interactions with the DHP backbone are evident from altered bond lengths. Several diagnostic bonds (N1(4)–N2(5), C5(9)–C6(10), and C7–C8) can typically be analyzed to probe redox state, but all of these bonds are significantly elongated in 1, putatively due to interaction with the K^+^ cation. However, the C7–C8 bond at the back of the pyrrole ring is most removed from the K^+^ cation and provides a useful metric to assay the electronic structure of the DHP ligand. Comparison of this distance in 1 to the Co(ii) DHP complexes, ([^*t*Bu,Tol^DHP]CoOTf) and ([^*t*Bu,Tol^DHP]Co(MeCN), suggests that the best oxidation state assignment for 1 is Co(iii) with a DHP ligand radical. A cyclic voltammogram of 1 shows additional oxidative features but we have not yet been able to isolate any oxidized complexes (Fig. S16, ESI†).

To confirm this formal oxidation state assignment, the electronic structure of 1 was further investigated with EPR spectroscopy (Fig. 2). The X-band EPR spectrum of 1 has a rhombic signal with features at *g*_*x*,*y*,*z*_ = 2.143, 2.015, 1.983 which are comparable to the signals of the parent hydroxide complex (Fig. S21, ESI†). The isotropic *g*-value for this complex is less deviated from the free-electron value than the starting complex (2.047 *vs.* 2.146) suggesting a smaller proportion of spin density localized at the Co center and consequently more DHP ligand radical character. The hyperfine coupling values support this assessment, as we observe larger coupling to N (^14^N *A*_*x*,*y*,*z*_ = 85.39, 87.94, 22.66 MHz) than to Co (^59^Co *A*_*x*,*y*,*z*_ = 37.85, 7.06, 22.66 MHz). These values sharply contrast with those of [^*t*Bu,Tol^DHP]Co(ii)OTf where the Co hyperfine couplings were larger than those for N.^27^ Further supporting the Co(iii) oxidation state assignment, these experimental Co < 38 MHz hyperfine couplings are similar to literature values for previously reported Co(iii)–superoxide complexes (isotropic ^59^Co hyperfine couplings of <45 MHz).^36^ Thus, the EPR data supports more ligand-centered radical character and a formal Co(iii) oxidation state.

**Fig. 2:**
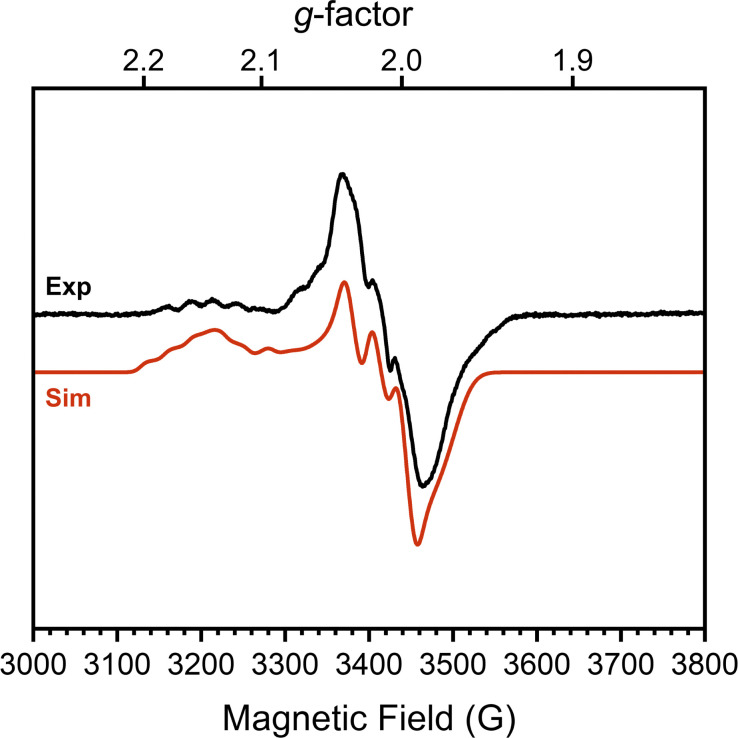
X-band EPR spectrum (black) and simulated spectrum (red) of a 15 mM solution of 1 (dimer) in toluene at 25 K. Conditions: MW frequency, 9.63 GHz; MW power, 2.0 mW. Simulation parameters: *g*_*x*,*y*,*z*_ = 2.143 2.015 1.983; ^59^Co *A*_*x*,*y*,*z*_ = 37.85 7.06 22.66 MHz; ^14^N *A*_*x*,*y*,*z*_ = 85.39, 87.94, 22.66 MHz; HStrain_*x*,*y*,*z*_ = 41.7975, 32.2248, 100.

Evans method analysis on complex 1 reveals *μ*_eff_ = 3.62*μ*_B_ per dimeric unit. This moment is consistent with either two *S* = 1/2 [^*t*Bu,Tol^DHP]CoOK fragments which are ferromagnetically coupled in the dimer or, possibly, two weakly coupled doublets. The *S* = 1/2 signal observed in EPR suggests that a weakly coupled pair of doublets is the most reasonable assignment, but some dissociation in solution is difficult to rule out. However, we note that all attempts to generate the monomer (*i.e.* with crown ethers) result in side reactivity and decomposition. This leads us to tentatively propose a weakly coupled dimer with the support of the EPR data.

Finally, the isolation of both 1 and [^*t*Bu,Tol^DHP]CoOH provides the possibility of determining the p*K*_a_ of the Co–OH unit. This p*K*_a_ is relevant to processes such as water oxidation, which has previously been observed in this system.^33^ We initially noted that neither 2,6-lutidine, used in previous studies with this system, nor NaOH, used in the formation of the Co–OH complex,^33^ result in deprotonation, although the poor solubility of NaOH in organic solvents complicates this conclusion. We undertook ^1^H NMR p*K*_a_ bracketing experiments using five weakly acidic alcohols. Protonation can be conveniently assayed by the appearance of a broad feature around 9.5 ppm, representative of the OH proton on the Co complex, which matches a feature present in the NMR spectrum of [^*t*Bu,Tol^DHP]Co(iii)OH (Fig. S2 and S8–S12, ESI†). These studies reveal that protonation of 1 occurs with hexafluoroisopropanol (HFIP), phenol and trifluoroethanol (TFE), but not with 2,4,6-tritertbutyl phenol (TTBP) or *tert*-butanol. From these results, the p*K*_a_ of 1 can be conservatively bracketed between TFE (p*K*_a_: 12.4 in water) and *tert*-butanol (p*K*_a_: 16.84 in water). A tighter bracket can be reasonably made with TTBP. However, we note that the p*K*_a_ of TTBP is not as well reported in water (p*K*_a_: ∼12.19, Table S7, ESI†). This bracketing tracks with single-point DFT calculations (Table S5, ESI†) and provides a useful general data point in examining the acidity of Co oxides and related species.

In summary, we report the synthesis of an unusual potassium capped terminal Co–oxido, [^*t*Bu,Tol^DHP]CoOK, 1. Complex 1 was characterized *via* SXRD to reveal a seesaw structure which is unlike similar crystallographically-characterized structures seen in the literature. Structural and spectroscopic analyses reveal that the electronic structure of 1 is best described as a weakly coupled dimer with Co(iii) metal centers and DHP ligand-based radicals. ^1^H NMR spectroscopy enables bracketing of the p*K*_a_ of this complex between 12.4 and 16.84 in water. The isolation of this unique compound expands how late metal–oxidos can accessed and stabilized. Further reactivity and oxidation studies would be an interesting avenue of investigation to examine the potential applications of [^*t*Bu,Tol^DHP]CoOK in oxidative reactivity.

This work was supported by the National Institutes of Health (R35GM133470). We also thank the Dreyfus foundation for a grant to J. S. A. (TC-21-064). We also thank the UChicago RCC for computing resources, and J. S. S. for EPR collection. We would also like to thank M. E. C., L. G., and M. W. for helpful discussions.

## Data availability

The data supporting this article have been included as part of the ESI.†

## Conflicts of interest

There are no conflicts to declare.

## Supplementary Material

CC-060-D4CC03014A-s001

CC-060-D4CC03014A-s002

CC-060-D4CC03014A-s003
