# Supplementary material for: Synthesis of a potassium capped terminal cobalt–oxido complex
Source: Chem Commun (Camb). 2024 Aug 8;60(71):9562–5. doi: 10.1039/d4cc03014a (PMC11327551; doi:10.1039/d4cc03014a)
Supplement: CC-060-D4CC03014A-s001 [file CC-060-D4CC03014A-s001.pdf]

Supplementary Information (SI) for ChemComm.
This journal is © The Royal Society of Chemistry 2024

Supplementary Information for:

Synthesis of a Potassium Capped Terminal Cobalt-Oxido Complex

Sophie W. Anferov,[a†] Alexandra Krupinski,[a†] John S. Anderson[a*]

[a]Department of Chemistry, University of Chicago, Chicago, Illinois 60637, United States.

[†]These authors contributed equally

*Corresponding author: jsanderson@uchicago.edu

Table of Contents

General Methods 3
Synthesis of [^{tBu,Tol}DHP]CoOK (1) 3
Characterization of [^{tBu,Tol}DHP]Co(MeCN) 3
Preparation of UV-Vis Samples 4
Preparation of IR Samples of (1) 4
Electrochemical Experiments 4
Density Functional Theory (DFT) Geometry Optimizations 4
NMR Spectroscopy 5
Figure S1. ^{1}H NMR of [$^{tBu, Tol}$DHP]CoOK (1) in C_6D_6. 5
Figure S2. Comparison of ^{1}H NMR of [$^{tBu, Tol}$DHP]CoOH in C_6D_6 (top) to ^{1}H NMR of [$^{tBu, Tol}$DHP]CoOK (1) in C_6D_6 (bottom). 6
Figure S3. 1H NMR of $(CF_3)_2CHOH$ (HFIP) in C_6D_6. 6
Figure S4. ^{1}H NMR of 2,4,6-tri-tert-butylphenol (2,4,6-TTBP) in C_6D_6. 7
Figure S6. ^{1}H NMR of Phenol in C_6D_6. 8
Figure S7. ^{1}H NMR of tert-butanol in C_6D_6 8
Figure S8. Comparison of ^{1}H NMR of 1 + HFIP in C_6D_6 (top) to 1 in C_6D_6 (bottom). 9
Figure S9. Comparison of ^{1}H NMR of 1 + TFE in C_6D_6 (top) to 1 in C_6D_6 (bottom) 9
Figure S10. Comparison of ^{1}H NMR of 1 + Phenol in C_6D_6 (top) to 1 in C_6D_6 (bottom) 10
Figure S11. Comparison of ^{1}H NMR of 1 + 2,4,6-TTBP in C_6D_6 (top) to 1 in C_6D_6 (bottom) 10
UV-Vis Spectroscopy 12
Figure S13. UV-vis of 1 from a 0.125 mM solution in toluene at room temperature. 12
IR Spectroscopy 12
Figure S14. IR of 1 as a thin film. 12
Figure S15. IR comparing IR of 1 (purple) as a thin film to IR of [$^{tBu, Tol}$DHP]CoOH (blue) as a thin film. 13
Cyclic Voltammetry 13
Figure S16. Cyclic Voltammogram of 2.4 mM of 1 in 0.1M KPF_6 electrolyte in a 1:4 MeCN:THF solution. Scan window: 1.5V to -3.5 V. 13
Figure S17. Cyclic Voltammogram of 2.4 mM of 1 in 0.1M KPF_6 electrolyte in a 1:4 MeCN:THF solution. Scan window: 0 V to -2.5 V 14
Single Crystal X-ray Diffraction 14
Figure S18. SXRD of 1. Co (pink), N (blue), C (gray), O (red), K(violet). C-H H-atoms omitted. 14
Table S1. SXRD of 1. 15
Table S2. Selected bond lengths (Å) and angles (°) of 1 and related Co complexes 15
Figure S19. SXRD of [^{tBu,Tol}DHP]Co(MeCN) . Co (pink), N (blue), C (gray), C–H H-atoms omitted for clarity. 16
Table S3. SXRD of [^{tBu,Tol}DHP]Co(MeCN) 16
EPR Spectroscopy 17
Figure S20. X-band EPR spectrum (black) and simulated spectrum (red) of a 15 mM solution of 1 (dimer) in toluene at 25 K. Conditions: MW frequency, 9.63 GHz; MW power, 2.0 mW. 17
Table S4. Simulated *g*-values for EPR of 1 17
Code to generate simulations of EPR Spectra for 1 18
Code to generate simulations of EPR Spectra for [$^{tBu, Tol}$DHP]CoOH. 18
Density Functional Theory (DFT) Calculations 19
Figure S22. Calculated structure of 1. All C–H hydrogen atoms have been removed for clarity 19
Figure S23. Spin density plot of 1 at an iso value of 0.005 19
Table S5. Single Point Energy Calculations of 1 with the addition of different acids 20
Figure S24. Calculated M–O bond lengths from monomeric (DHP)CoOM complexes (S = ½) where M is each alkali metal (going down column 1 of the periodic table, rows 1-7). 20

Table S6. Single Point Energy Calculations of 1 with different alkali metal cations 20
pK_a Values of Weak Acids... 20
Table S7. pK_a values for acids used in pK_a bracket study 20
References 21

General Methods

All chemicals were purchased from commercial suppliers and used without further purification. All manipulations were carried out under an atmosphere of N_2 using standard Schlenk and glovebox techniques. Glassware was dried at 180 °C for a minimum of two hours and cooled under vacuum prior to use. Solvents were dried on a solvent purification system from Pure Process Technologies and stored over 4 Å molecular sieves under N_2. Tetrahydrofuran (THF) was stirred over NaK alloy and run through an additional alumina column prior to use to ensure dryness. Solvents were tested for H_2O and O_2 using a standard solution of sodium-benzophenone ketyl radical anion. CD_3CN, C_6D_6, and d_8-toluene were dried over 4 Å molecular sieves under N_2. ^1H NMR spectra were recorded on Bruker DRX 400 or 500 spectrometers. Chemical shifts are reported in ppm units referenced to residual solvent resonances for ^1H NMR spectra. UV-visible spectra were recorded on a Bruker Evolution 300 spectrometer and analyzed using VisionPro software. A standard 1 cm quartz cuvette with an airtight screw cap with a puncturable Teflon seal was used for all measurements. A Unisoku CoolSpek cryostat was used for low temperature measurements. IR spectra were recorded on a Bruker Tensor II spectrometer with the OPUS software suite as DCM thin films between KBr plates. Single crystal X-ray diffraction data were collected in-house using Bruker D8 Venture diffractometer equipped with Mo microfocus X-ray tube (λ = 0.71073 Å). Electrochemical measurements were carried out using a BAS Epsilon potentiostat and using BAS Epsilon software version 1.40.67 NT. EPR spectra were recorded on an Elexsys E500 spectrometer with an Oxford ESR 900 X-band cryostat and a Bruker Cold-Edge Stinger and were simulated using the Easyspin suite in Matlab software.[1] Magnetic moments were determined using the Evans method.[2]

*Synthesis of [^{tBu,Tol}DHP]CoOK (**1**)*

In a 20 mL vial in the glovebox, 2 mL of toluene was added to [^{tBu,Tol}DHP]CoOH (0.002 g, 1 eq., 0.004 mmol).[3] A suspension of potassium tert-butoxide (0.0022 g, 5 eq., 0.020 mmol) in toluene was added to the bright purple solution of [^{tBu,Tol}DHP]CoOH. After stirring for 1-2 h, or until the suspended white solids were no longer visible, the resulting green-purple solution was dried *in vacuo* and extracted into benzene. Complex **1** can then be isolated as a purple solid after crystallizations in petroleum ether. Yield: 0.0019 g, 88 %. Poor quality single crystals suitable for XRD of **1** were grown out of a cooled concentrated petroleum ether solution at -35°C. ^1H NMR (400 MHz, C_6D_6, RT): δ = 10.92 (br s), 8.44 (s), 6.67 (s), 1.88(s), 1.50(s). Magnetic Susceptibility: Evans' Method for **1** (C_7D_8 RT, 500 MHz, μ_B): μ_{eff} = 3.62, UV-vis, nm in toluene, (ε, $M^{-1}cm^{-1}$): 2213.32. HRMS (EI) m/z: $[M]^+$ calculated for **1**: $C_{28}H_{34}N_5OKCo$ 554.1732 found: 554.1775.

In practice, this compound can also be obtained from [^{tBu,Tol}DHP]CoCl or OTf via addition of wet KO^{t}Bu.[4]

Characterization of [^{tBu,Tol}DHP]Co(MeCN)

The [^{tBu,Tol}DHP]Co(MeCN) has been previously reported.[5] In this work we report its crystal structure.

Preparation of UV-Vis Samples

An aliquot of complex **1** was dissolved in toluene in a quartz cuvette in the glove box. The 1 cm quartz cuvette was equipped with an airtight screw cap. The spectrum was collected under a blanketing flow of Nitrogen.

*Preparation of IR Samples of (**1**)*

Separate samples of complex **1** and [$^{tBu, Tol}$DHP]CoOH were dissolved in dry dichloromethane to form a concentrated solution. This was dropcast onto a KBr plate, and a second plate was then placed on top. The sample was then transferred in an air-free temporary container to the spectrometer, and a spectrum was collected.

Electrochemical Experiments

Experiments were performed inside the glovebox with a 1:4 MeCN:THF 0.1 M KPF_6 electrolyte solution at room temperature. Cyclic voltammetry measurements were made with a [Co] = 2.4 mM using a glassy carbon working electrode, platinum wire counter electrode, and silver wire pseudo reference electrode and were referenced to internal Fc/Fc+ by adding ferrocene at the end of measurements. A one-compartment glass cell was filled with 4 mL of electrolyte solution. The working electrode was polished over a microcloth pad (Buehler) using alumina slurry (0.05mm EMS), followed by rinsing with deionized water and isopropyl alcohol. Reference and counter electrodes were rinsed with acetone. CVs were recorded at a scan rate of 200 mV/s scanning oxidatively.

Density Functional Theory (DFT) Geometry Optimizations

Geometry optimization calculations and single point energy calculations were performed with ORCA[6] software suite using density functional theory (DFT). Geometries were fully optimized starting from coordinates generated from finalized cifs of the compound crystal structures when possible. The O3LYP functional was used for geometry optimizations, spin density plot calculations, and single point energy calculations. For the O3LYP calculations, def2-TZVPP was used on Co, N, S, O, and F, and def2-TZVP on C and H atoms. A CPCM solvation model for benzene was used throughout. Due to the challenge of simulating the dimeric OK complex, the calculation energies here do not include entropic contributions.

NMR Spectroscopy

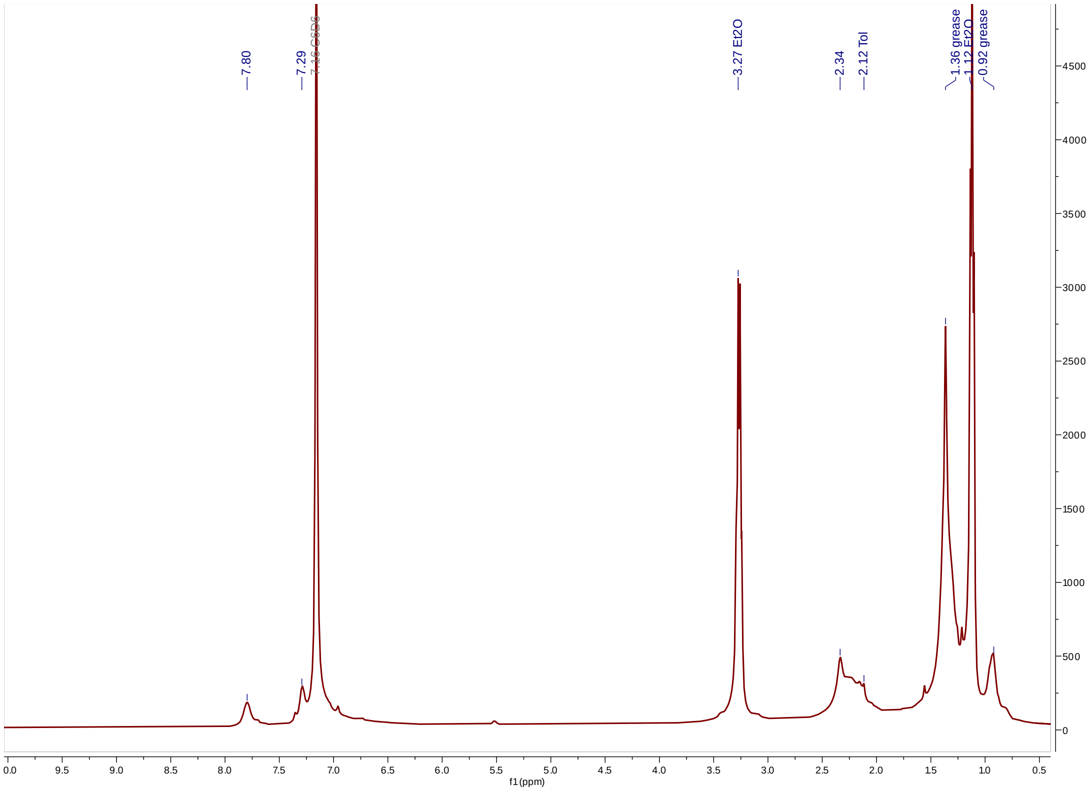

Figure S1. ^{1}H NMR of [$^{t\text{Bu, Tol}}$DHP]CoOK (**1**) in C_6D_6.

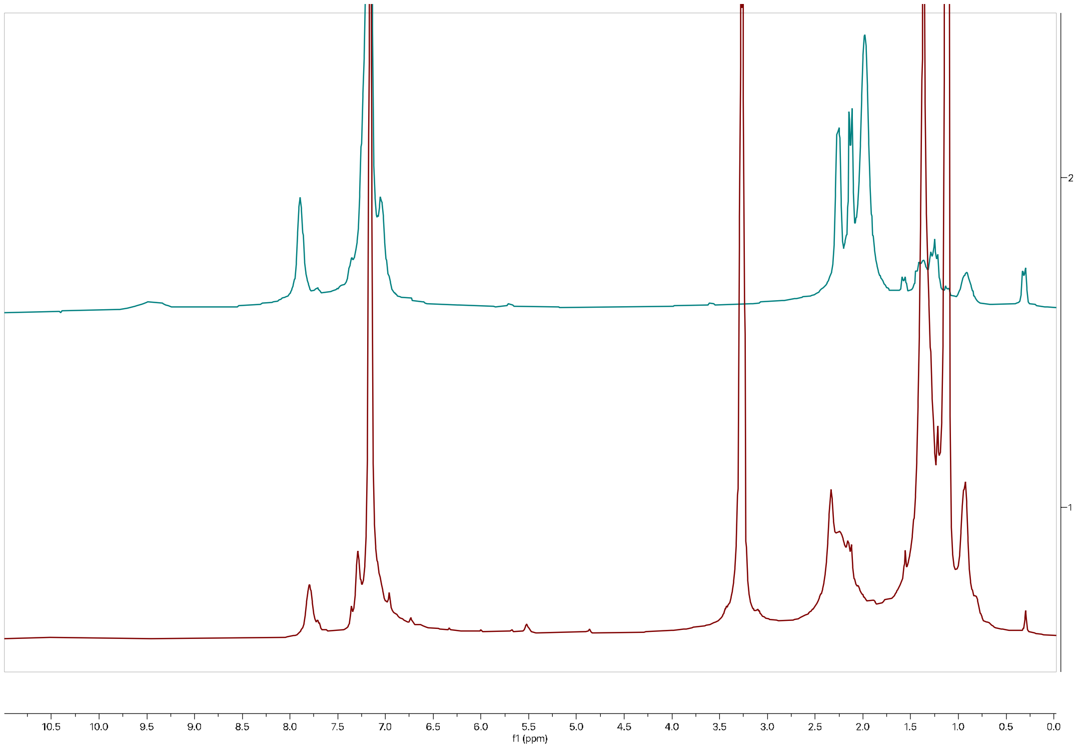

Figure S2. Comparison of ^{1}H NMR of $[^{tBu,\,Tol}DHP]CoOH$ in C_6D_6 (top) to ^{1}H NMR of $[^{tBu,\,Tol}DHP]CoOK$ (**1**) in C_6D_6 (bottom).

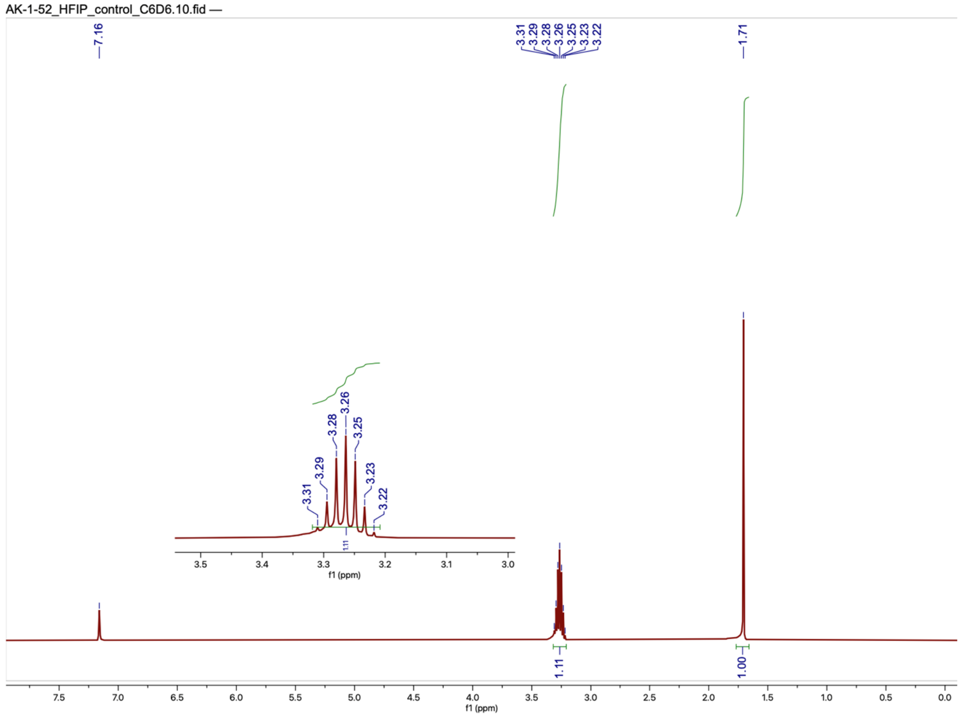

Figure S3. 1H NMR of $(CF_3)_2CHOH$ (HFIP) in C_6D_6.

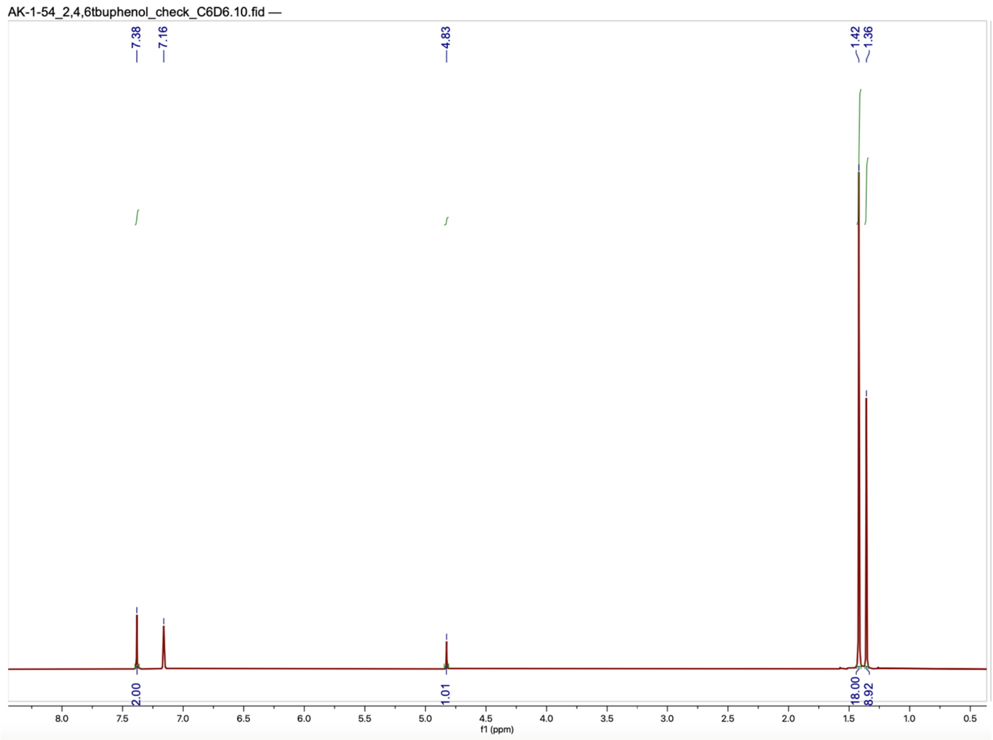

Figure S4. ^{1}H NMR of 2,4,6-tri-tert-butylphenol (2,4,6-TTBP) in C_6D_6.

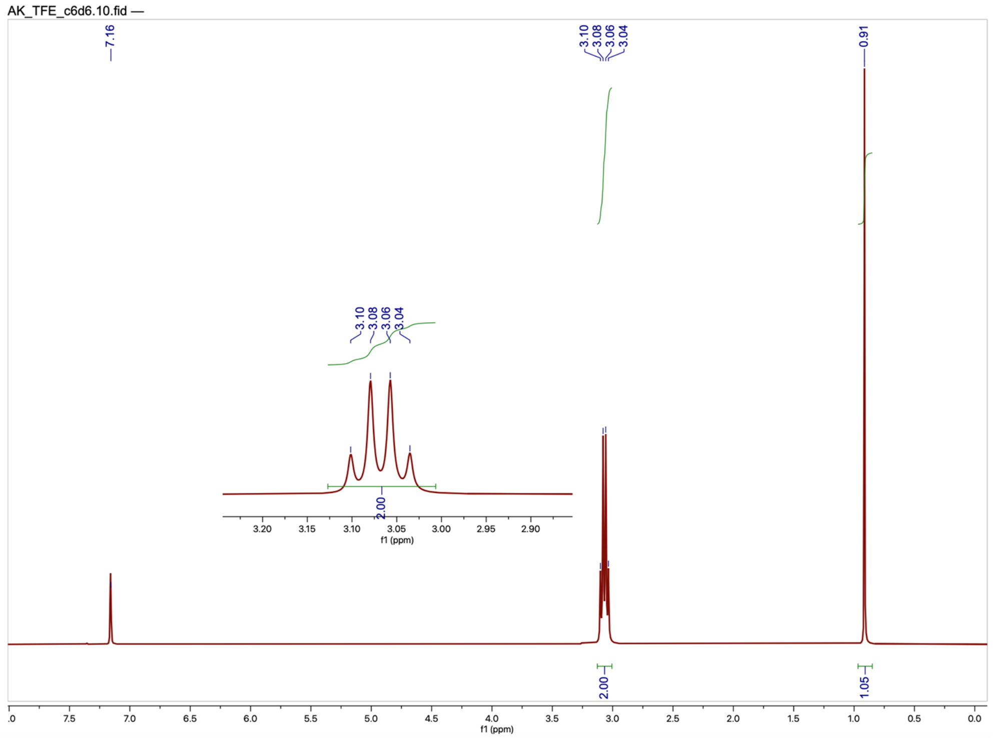

Figure S5. ^{1}H NMR of 2,2,2-Trifluoroethanol (TFE) in C_6D_6.

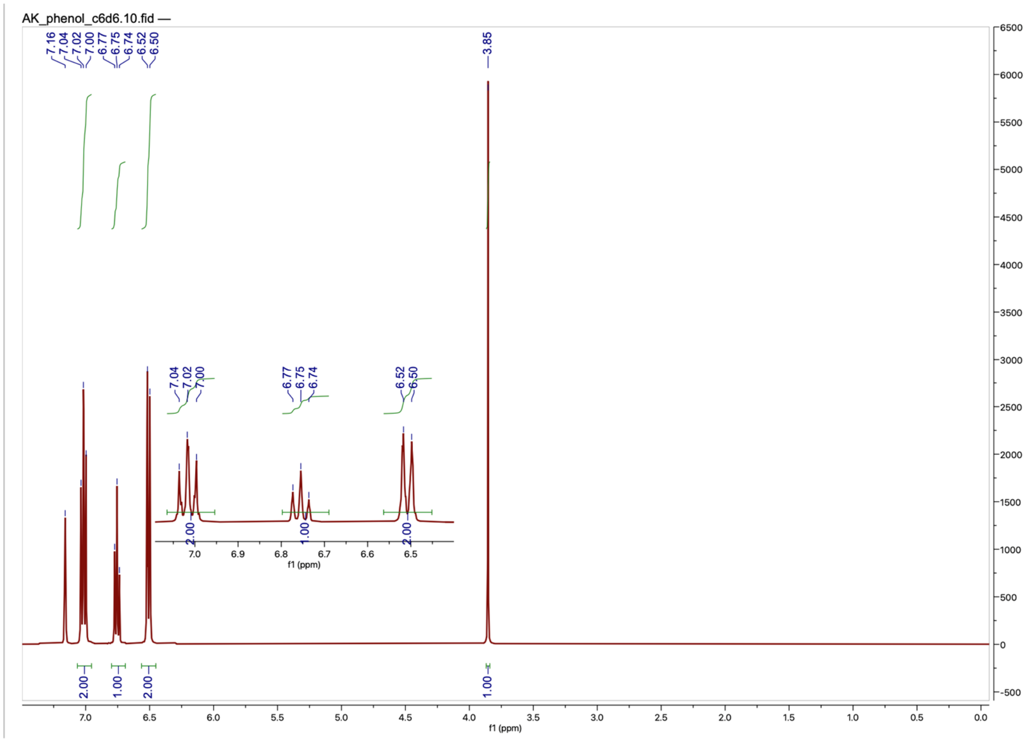

Figure S6. ^{1}H NMR of Phenol in C_6D_6.

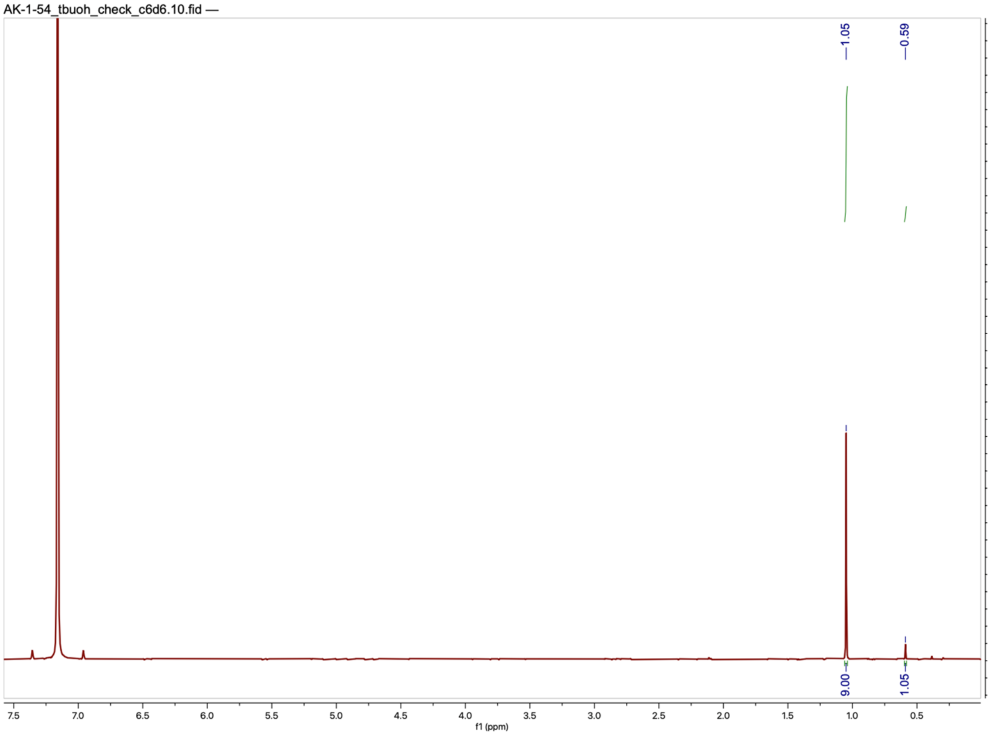

Figure S7. ^{1}H NMR of tert-butanol in C_6D_6

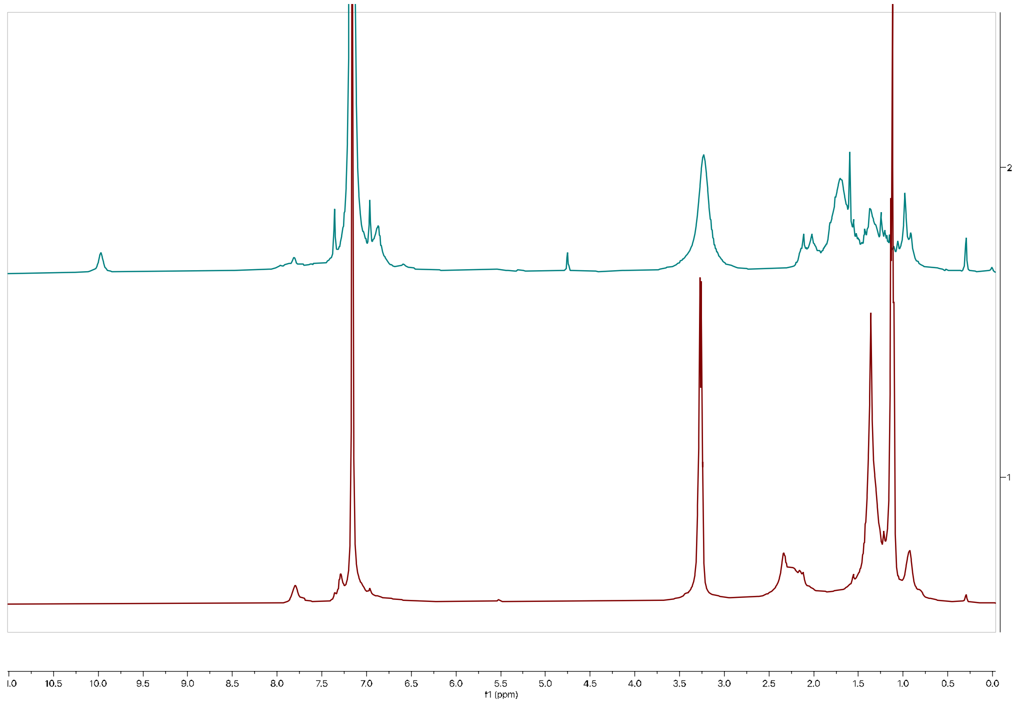

Figure S8. Comparison of ^{1}H NMR of **1** + HFIP in C_6D_6 (top) to **1** in C_6D_6 (bottom)

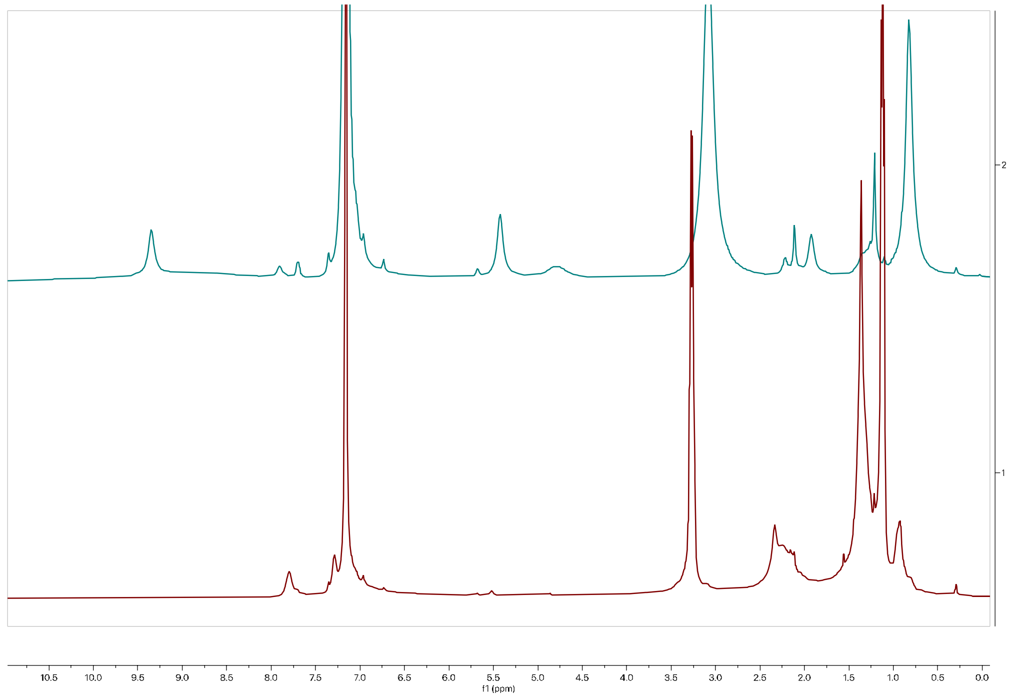

Figure S9. Comparison of ^{1}H NMR of **1** + TFE in C_6D_6 (top) to **1** in C_6D_6 (bottom)

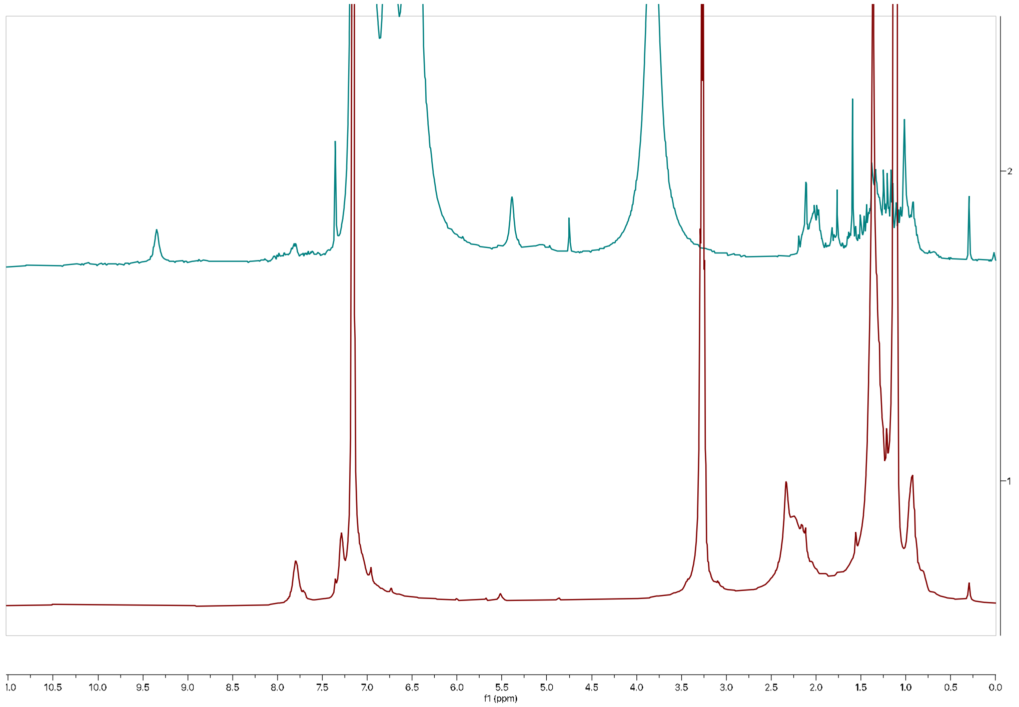

Figure S10. Comparison of ^{1}H NMR of **1** + Phenol in C_6D_6 (top) to **1** in C_6D_6 (bottom)

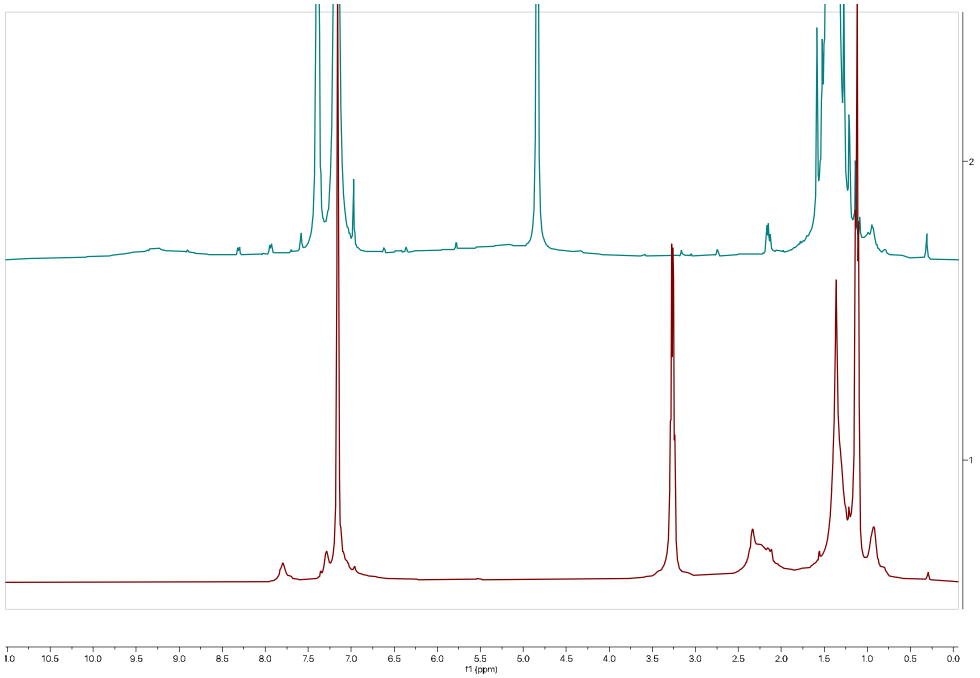

Figure S11. Comparison of ^{1}H NMR of **1** + 2,4,6-TTBP in C_6D_6 (top) to **1** in C_6D_6 (bottom)

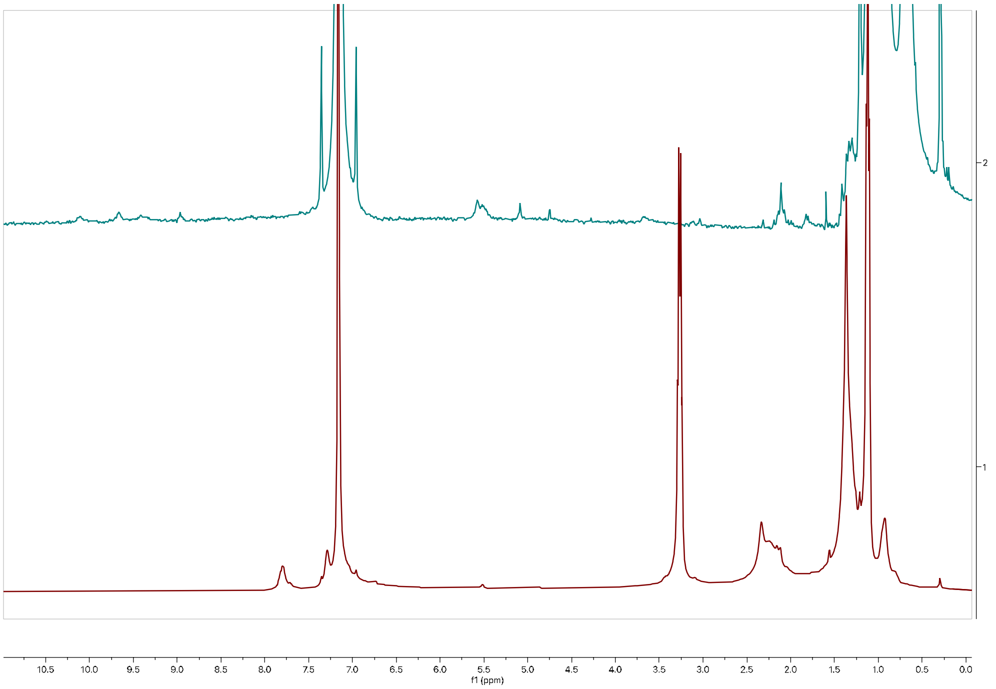

Figure S12. Comparison of ^{1}H NMR of **1** + tert-Butanol in C_6D_6 (top) to **1** in C_6D_6 (bottom)

UV-Vis Spectroscopy

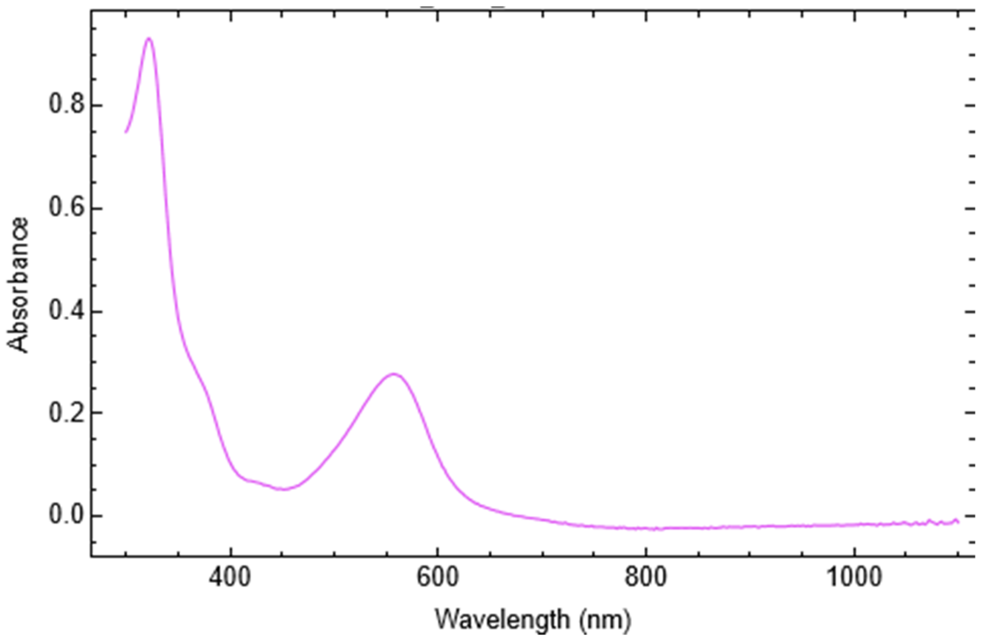

Figure S13. UV-vis of **1** from a 0.125 mM solution in toluene at room temperature.

IR Spectroscopy

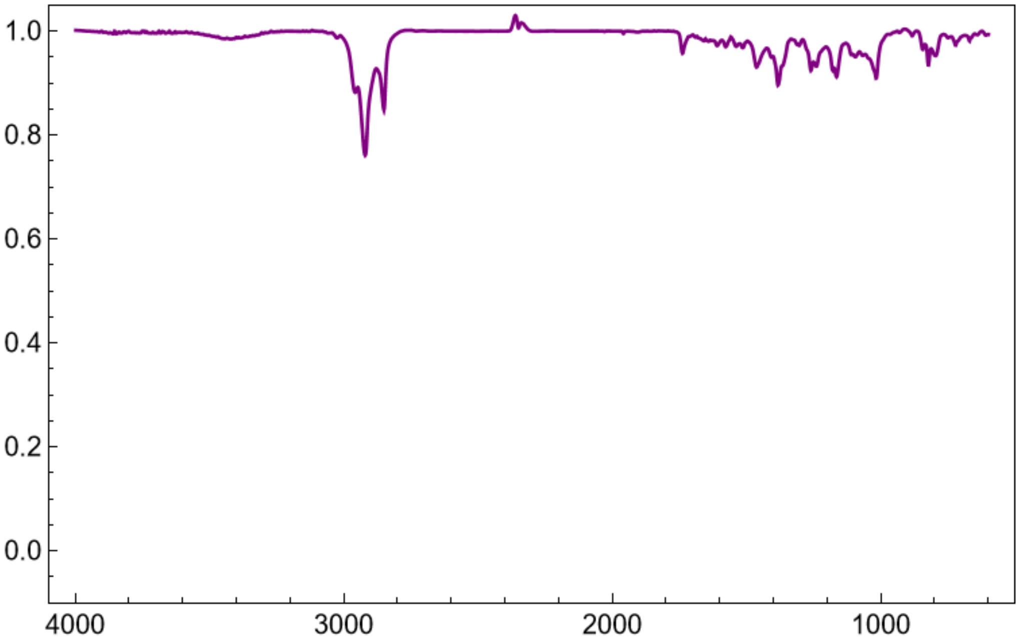

Figure S14. IR of **1** as a thin film.

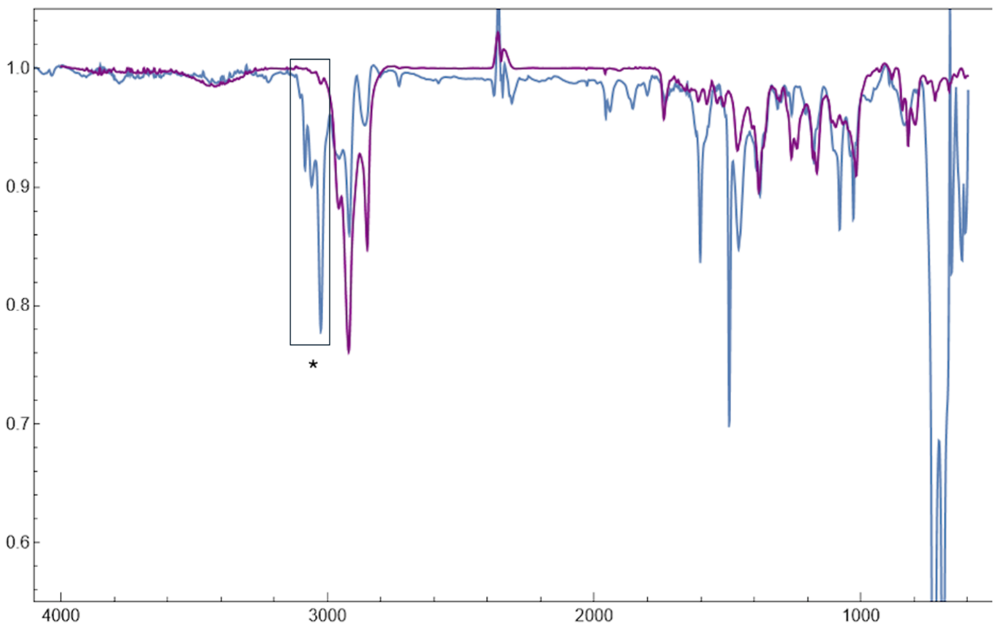

Figure S15. IR comparing IR of **1** (purple) as a thin film to IR of [$^{tBu, Tol}$DHP]CoOH (blue) as a thin film. The OH peak of [tBu, TolDHP]CoOH is marked by an asterisk (*) and is generally consistent DFT calculated values.3 However, we note a few overlapping stretches, presumably from C–Hs. The low frequency of this OH made arise from hydrogen bonding interactions between molecules.

Cyclic Voltammetry

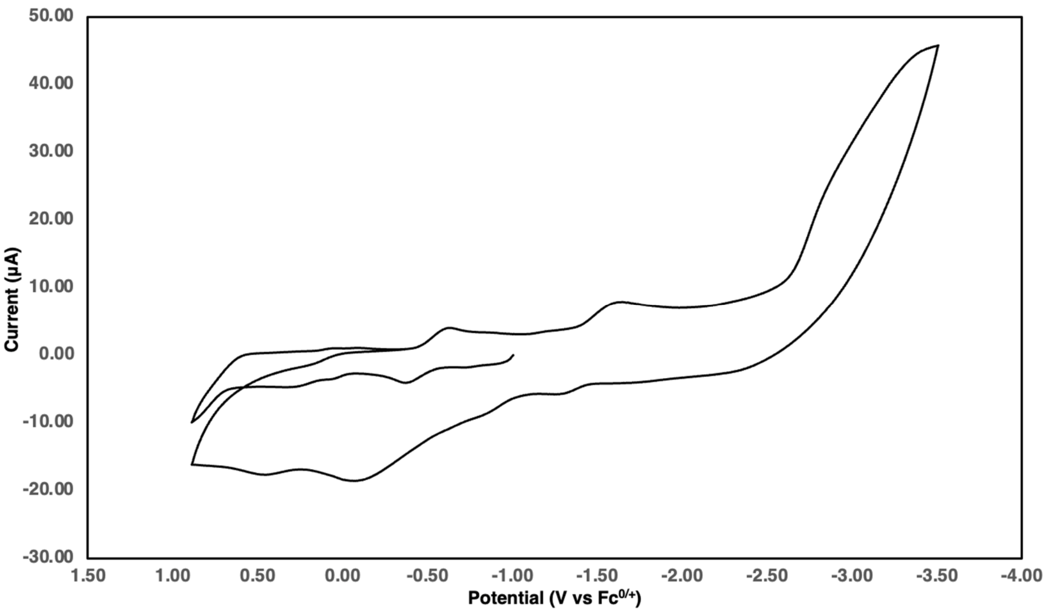

Figure S16. Cyclic Voltammogram of 2.4 mM of **1** in 0.1M KPF_6 electrolyte in a 1:4 MeCN:THF solution. Scan window: 1.5V to -3.5 V.

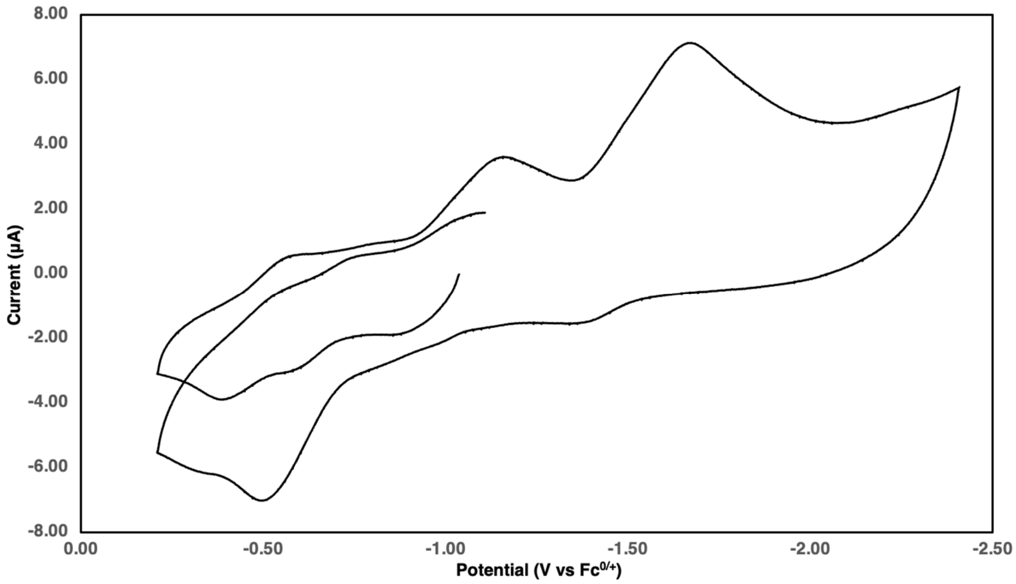

Figure S17. Cyclic Voltammogram of 2.4 mM of **1** in 0.1M KPF_6 electrolyte in a 1:4 MeCN:THF solution. Scan window: 0 V to -2.5 V

Single Crystal X-ray Diffraction

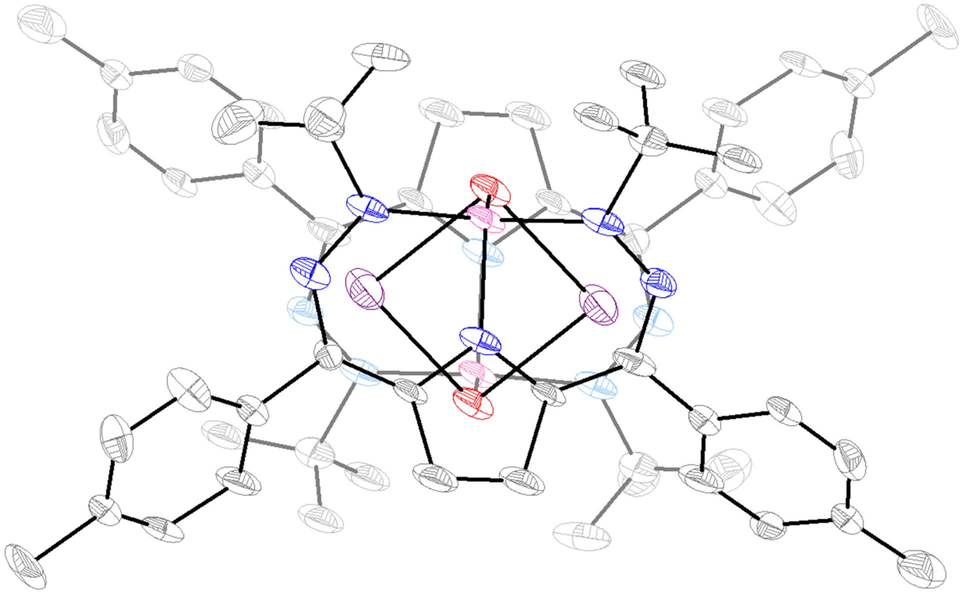

Figure S18. SXRD of **1**. Co (pink), N (blue), C (gray), O (red), K(violet). C-H H-atoms omitted.

Selected bond lengths (Å): Co-N1/N5: 1.94(1), 1.93(1); Co-N3: 1.94(1); N1-N2/N4-N5: 1.38(2), 1.32(2); Co-O: 1.96(1); N2-C5/N4-C10: 1.35(2), 1.33(2); C5-C6/C9-C10: 1.43(2), 1.41(2); C6-C7/C8-C9: 1.44(2), 1.42(2); C7-C8: 1.38(2). Selected bond angles (°): N1-M-N5: 149.4(6); N3-M-O: 107.4(5).

Table S1. SXRD of **1**.

Empirical formula	$C_{28}H_{34}CoKN_5O$
Formula weight	554.63
Temperature/K	100(2)
Crystal system	triclinic
Space group	P-1
a/Å	10.153(7)
b/Å	11.686(8)
c/Å	13.651(10)
α/°	89.533(19)
β/°	73.197(17)
γ/°	64.696(15)
Volume/Å^3	1389.1(17)
Z	2
ρ_{calc}g/cm^3	1.326
μ/mm^{-1}	0.797
F(000)	582.0
Crystal size/mm^3	0.242 × 0.072 × 0.052
Radiation	MoKα (λ = 0.71073)
2Θ range for data collection/°	4.566 to 46.918
Index ranges	$-11 \le h \le 11$, $-12 \le k \le 13$, $-15 \le l \le 15$
Reflections collected	14083
Independent reflections	4025 [R_{int} = 0.2607, R_{sigma} = 0.2678]
Data/restraints/parameters	4025/216/261
Goodness-of-fit on F^2	1.115
Final R indexes [I>=2σ (I)]	R_1 = 0.1435, wR_2 = 0.3264
Final R indexes [all data]	R_1 = 0.2880, wR_2 = 0.4058
Largest diff. peak/hole / e Å^{-3}	1.76/-0.99

Table S2. Selected bond lengths (Å) and angles (°) of **1** and related Co complexes

	**DHPCoOK**	**[DHP^{2-}]Co^{II}(MeCN)$^-$**	**[DHP^{1-}]CoOTf**	**[DHP^{2-}]Co^{III}OH**
M—N1/M—N5	1.96(1)<br>1.92(1)	1.936(3)<br>1.938(3)	1.999(8)<br>2.000(8)	1.897(3)<br>1.889(3)
M—N3	1.94(1)	1.938(3)	1.918(7)	1.861(3)
N1—N2/ N4—N5	1.38(2)<br>1.32(2)	1.325(5)<br>1.320(5)	1.273(10)<br>1.249(10)	1.306(3)<br>1.302(3)
M—X(Cl/O/N)	1.96(1)	2.076(4)	2.166<br>2.128(6)	1.825(2)
N2—C5/ N4—C10	1.35(2)<br>1.33(2)	1.344(6)<br>1.339(6)	1.368(11)<br>1.383(11)	1.350(4)<br>1.342(4)
C5—C6/ C9—C10	1.44(2)<br>1.42(2)	1.402(6)<br>1.399(6)	1.387(13)<br>1.392(13)	1.390(4)<br>1.383(4)

C6—C7 / C8—C9	1.44(2)<br>1.41(2)	1.441(6)<br>1.417(6)	1.428(13)<br>1.445(13)	1.439(4)<br>1.438(4)
C7—C8	1.39(2)	1.351(6)	1.315(13)	1.349 (4)
N1—M—N5	149.1(5)	160.51(12)	178.1(3)	162.4(1)
N3—M—X	107.4(5)	114.32(13)	113.152<br>105.240	143.5(1)

L = PMe_3 as described in the text.

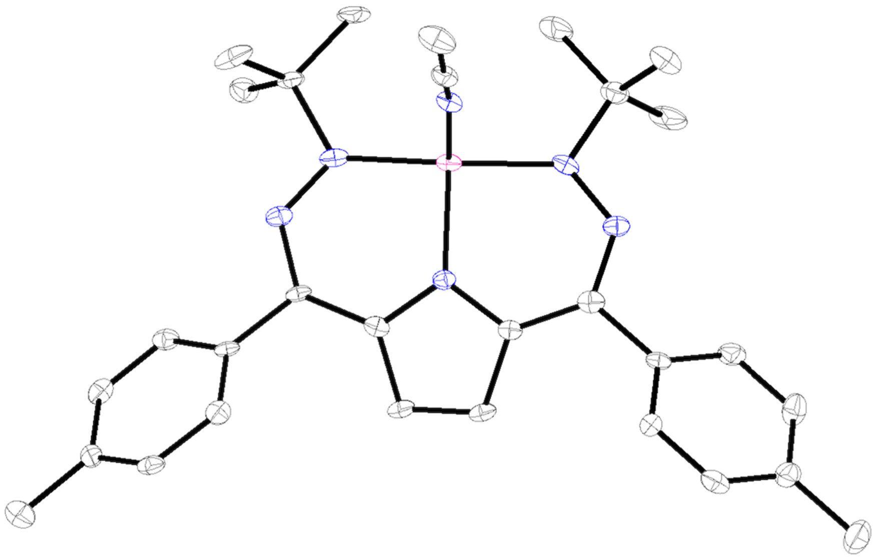

Figure S19. SXRD of [^{tBu,Tol}DHP]Co(MeCN) . Co (pink), N (blue), C (gray), C–H H-atoms omitted for clarity.

Table S3. SXRD of [^{tBu,Tol}DHP]Co(MeCN)

Empirical formula	$C_{30}H_{37}CoN_6$
Formula weight	540.58
Temperature/K	100(2)
Crystal system	orthorhombic
Space group	$P2_12_12_1$
a/Å	8.9652(6)
b/Å	11.2879(7)
c/Å	27.8086(17)
α/°	90
β/°	90
γ/°	90
Volume/Å^3	2814.2(3)
Z	4
ρ_{calc}g/cm^3	1.276
μ/mm^{-1}	0.639

F(000)	1144.0
Crystal size/mm^3	0.297 × 0.122 × 0.059
Radiation	MoKα (λ = 0.71073)
2Θ range for data collection/°	4.648 to 50.264
Index ranges	-10 ≤ h ≤ 10, -13 ≤ k ≤ 13, -33 ≤ l ≤ 33
Reflections collected	54762
Independent reflections	5025 [R_{int} = 0.1142, R_{sigma} = 0.0647]
Data/restraints/parameters	5025/0/337
Goodness-of-fit on F^2	1.061
Final R indexes [I>=2σ (I)]	R_1 = 0.0459, wR_2 = 0.0727
Final R indexes [all data]	R_1 = 0.0709, wR_2 = 0.0791
Largest diff. peak/hole / e Å^{-3}	0.38/-0.36

EPR Spectroscopy

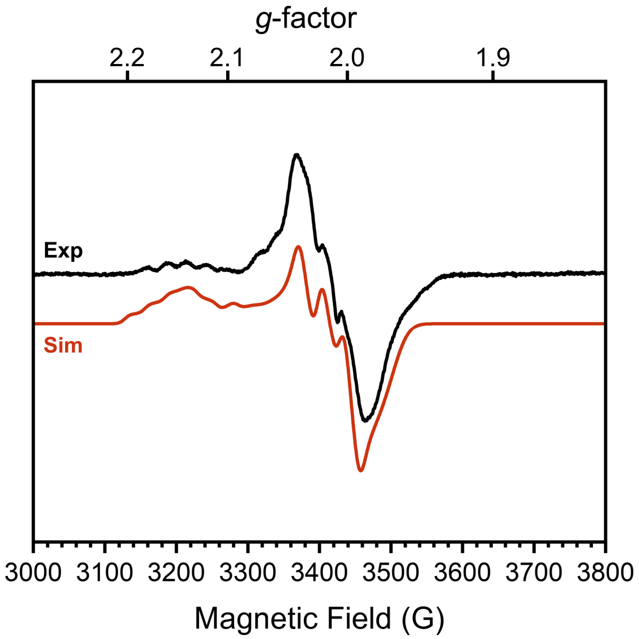

Figure S20. X-band EPR spectrum (black) and simulated spectrum (red) of a 15 mM solution of **1** (dimer) in toluene at 25 K**.** Conditions: MW frequency, 9.63 GHz; MW power, 2.0 mW.

Table S4. Simulated *g*-values for EPR of **1**

	g (x,y,z)	Co-A	N-A	H-strain
^{tBu}DHPCoOK	(2.14553 2.015 1.98313)	(37.85 7.0606 22.664)	(85.3902 87.9404 22.6612)	(41.7975 32.2248 100)

Code to generate simulations of EPR Spectra for **1**.

```
Exp1.mwFreq=9.63;
Sys1.lw = 1
Exp1.Range = [300 380]
Exp1.nPoints = 2048
Sys1.g = [2.14553 2.015 1.98313]
Sys1.Nucs = 'Co,N';
Sys1.A = [37.85 7.0606 22.664;85.3902 87.9404 22.6612];
Sys1.HStrain = [41.7975 32.2248 100];
Vary1.g = [0.02 0.02 0.02];
Vary1.HStrain = [20 1.5 50];
Vary1.A = [10 10 10; 10 10 10];
plot(Bk,(((Ik)/max(Ik))0.225)*2,Bk,pepper(Sys1,Exp1)/(max(pepper(Sys1,Exp1)))
)
```

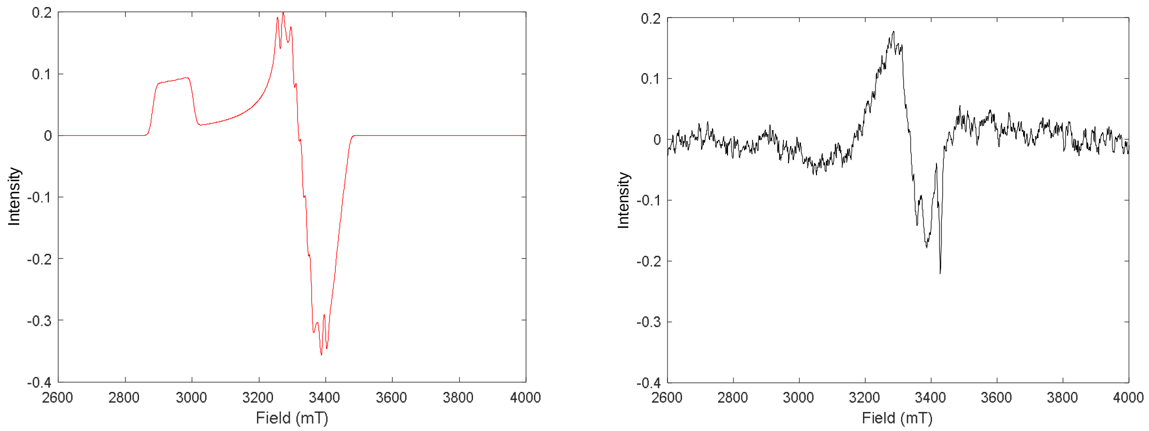

Figure S21. Small Window perpendicular-mode EPR spectrum (right) and simulated spectrum (left) of a 15 mM solution of [$^{t\mathrm{Bu,\ Tol}}$DHP]CoOH in toluene at 25 K. Conditions: MW frequency, 9.63 GHz; MW power, 2.0 mW.

Code to generate simulations of EPR Spectra for [$^{t\mathrm{Bu,\ Tol}}$DHP]CoOH.

```
Exp3.mwFreq=9.63913;
Sys3.lw = 1
Exp3.Range = [260 400]
Exp3.nPoints = 2048;
Sys3.g = [2.34 2.068 2.03];
Sys3.Nucs = 'Co, N';
Sys3.A = [50 40 40; 20 60 50];
Sys3.HStrain = [50 10 50];
Vary3.g = [0.02 0.02 0.02];
Vary3.HStrain = [50 10 50];
Vary3.A = [10 10 10; 10 10 10];
plot(BOH,(IOH)/max(IOH)-(-
0.000086*BOH+1.1044),BOH,0.2*pepper(Sys3,Exp3)/max(pepper(Sys3,Exp3)))
```

Density Functional Theory (DFT) Calculations

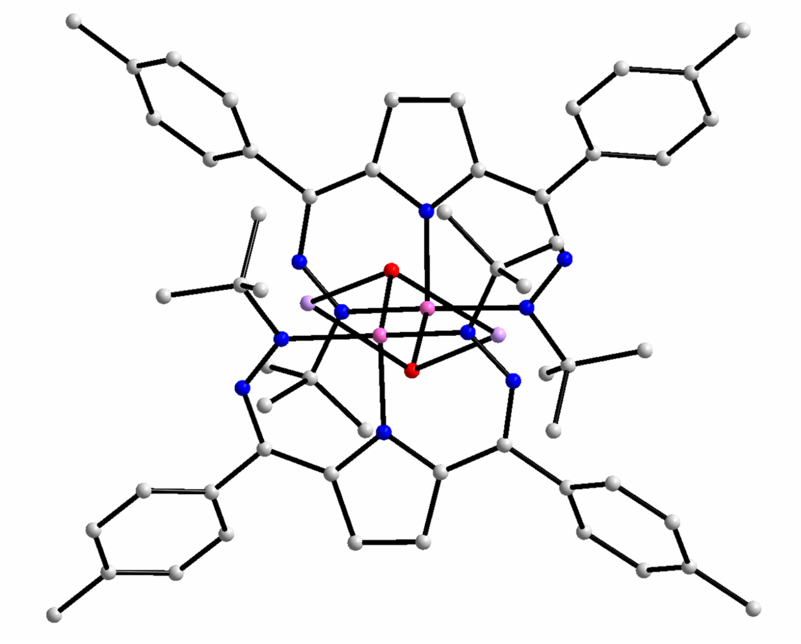

Figure S22. Calculated structure of **1**. All C–H hydrogen atoms have been removed for clarity

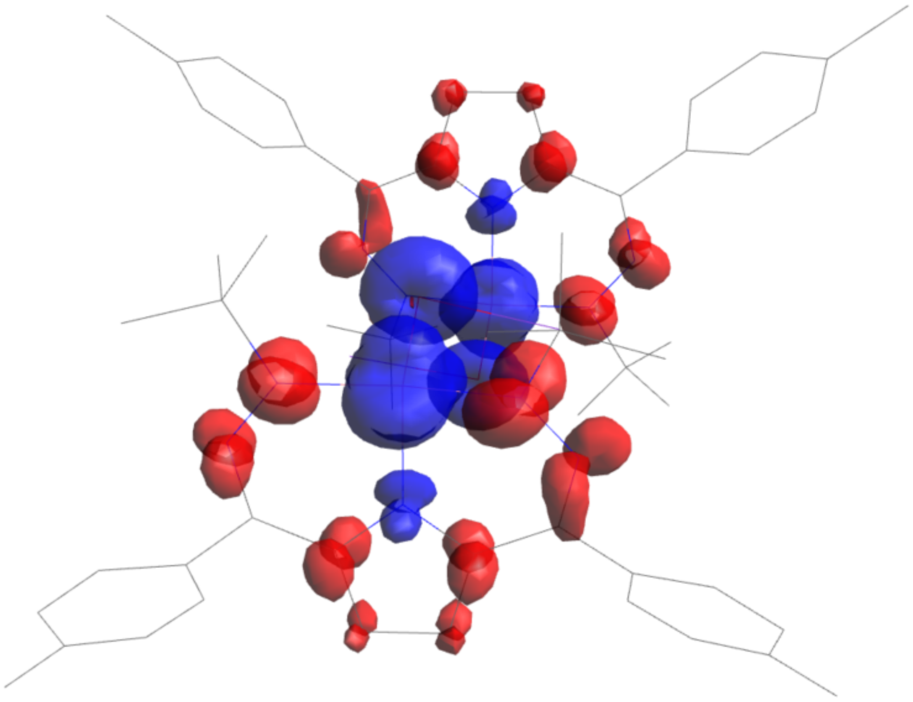

Figure S23. Spin density plot of **1** at an iso value of 0.005.

Table S5. Single Point Energy Calculations of **1** with the addition of different acids

Dimer	From LCoOH+ K[acid]	LCoOK+ tbuOH	LCoOK+ CyOH	LCoOK+ IPA	LCoOK+ acetamide	LCoOK+ TFE	LCoOK+ Phenol	LCoOK+ 2,4,6-TTBP	LCoOK+ PhenSL	LCoOK+ phthalamide	LCoOK+ HFIP	LCoOK+ BzCOOH	LCoOK dimer
Kcal/ mol		-18.01	-17.82	-17.19	-7.13	-6.05	-2.98	-2.63	-1.62	-0.80	1.77	12.48	-14.75
Monomer		LCoOK+ tbuOH	LCoOK+ CyOH	LCoOK+ IPA	LCoOK+ acetamide	LCoOK+ TFE	LCoOK+ Phenol	LCoOK+ 2,4,6-TTBP	LCoOK+ PhenSL	LCoOK+ phthalamide	LCoOK+ HFIP	LCoOK+ BzCOOH	All single point calculations (no entropy)
Kcal/ mol		-3.26	-3.07	-2.44	7.63	8.71	11.77	12.12	13.14	13.96	16.52	27.23	

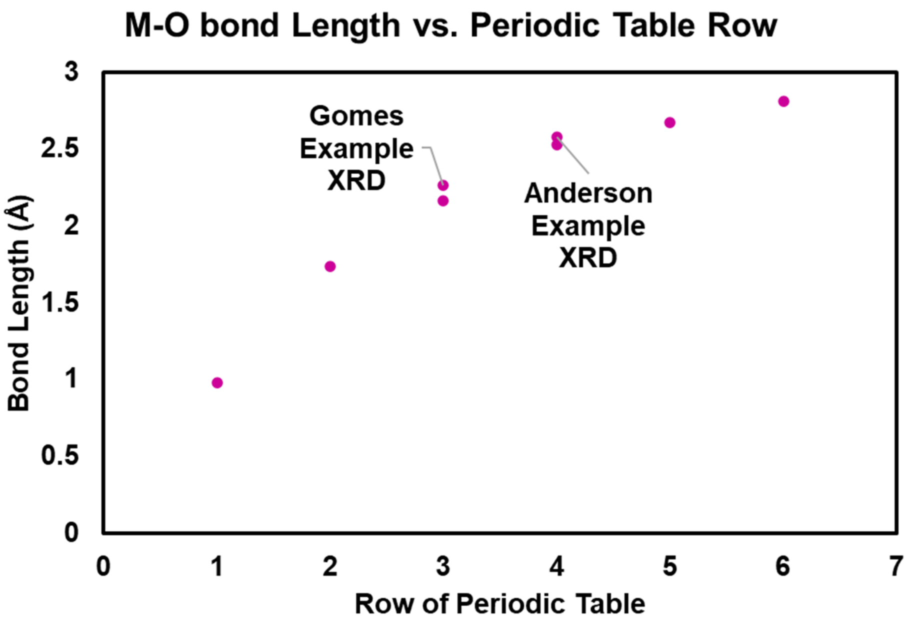

Figure S24. Calculated M–O bond lengths from monomeric (DHP)CoOM complexes (S = ½) where M is each alkali metal (going down column 1 of the periodic table, rows 1-7).

Table S6. Single Point Energy Calculations of **1** with different alkali metal cations

Bond lengths in Å	LCoOH	LCoOLi	LCoONa	LCoOK	LCoORb	LCoOCs	LCoOK SXRD	Gomes SXRD	hydroxide
single O-M bond	0.976	1.738	2.164	2.53	2.675	2.808	2.58	2.263	--

pK_a Values of Weak Acids

Table S7. pK_a values for acids used in pK_a bracket study

pK_a	tbuOH	2,4,6-TTBP	TFE	Phenol	HFIP
Water	16.84[7]	12.19[8]	12.4[9]	10.0[10]	9.3[11]
DMSO	29.4[13]	--	23.6[9]	18.0[9]	18.2[15]
MeCN	--	--	35.8 (calc.)[14]	29.2[12]	--

References

1. S. Stoll and A. Schweiger, *J. Magn. Reson*., 2006, **178**, 42−55.
2. D.F. Evans, *J. Chem. Soc.*, 1959, **0**, 2003-2005.
3. S.W. Anferov and J.S. Anderson, *J. Coord. Chem.*, 2022, **75**, 1853-1864.
4. S.W. Anferov, A.S. Filatov and J.S. Anderson, *ACS Catal.*, 2022, **12**, 9933-9943.
5. S.W. Anferov, J. Boyn, D.A. Mazziotti, J.S. Anderson, *J. Am. Chem. Soc.*, 2024, **146**, 5855-5863.
6. (a) F. Neese, *Wiley Interdis. Rev.: Comp. Mol. Sci.*, 2012, **2**, 73-78. (b) H - Kr: A. Schaefer, H. Horn and R. Ahlrichs, *J. Chem. Phys*., 1992, **97**, 2571. (c) Rb - Xe: A.Schaefer, C. Huber and R. Ahlrichs, *J. Chem. Phys.*, 1994, **100**, 5829. (d) F. Weigend and R. Ahlrichs, *Phys. Chem. Chem. Phys.* 2005, **7**, 3297
7. A.S. Tracey, B. Galeffi and S. Mahjour, *Can. J. Chem.*, 1988, **66**, 2294 – 2298.
8. E. P. Serjeant and B. Dempsey, *Ionization Constants of Organic Acids in Solution*, IUPAC Chemical Data Series No. 23,1979.
9. F.G. Bordwell, *Acc. Chem. Res.*, 1988, **21**, 456-463
10. M. Walton-Raaby, T. Floen, G. García-Díez, and N. Mora-Diez, *Antioxidants*, 2023, **12**, 1420.
11. B.L. Dyatkin, E.P. Mochalina, and I.L. Knunyants, *Tetrahedron,* 1965, **21,** 2991-2995.
12. A. Kütt, S. Tshepelevitsh, J. Saame, M. Lõkov, I. Kaljurand, S. Selberg, and I. Leito, *Eur. J. Org. Chem*., 2021, **2021,** 1305−1473
13. W.N. Olmstead, Z. Margolin and F.G. Bordwell, *J. Org. Chem.*, 1980, **45**, 3295-3299.
14. Y.C. Lam, R.J. Nielsen, H.B. Gray and W.A. Goddard, *ACS Catal.*, 2015, **5**, 2521-2528.
15. D. Seebach, A.K. Beck, H. Bichsel, A. Pichota, C. Sparr, R. Wünsch and W.B. Schweizer, *Helv. Chim. Acta*, 2012, **95**, 1303-1322.
